# A Face-Shear Mode Piezoelectric Array Sensor for Elasticity and Force Measurement

**DOI:** 10.3390/s20030604

**Published:** 2020-01-22

**Authors:** Kyungrim Kim, Taeyang Kim, Jinwook Kim, Xiaoning Jiang

**Affiliations:** 1 Department of Mechanical and Aerospace Engineering, North Carolina State University, Raleigh, NC 27695, USA; kkim8@ncsu.edu; 2 Department of Mechanical and System Engineering, Korea Military Academy, Seoul 01805, Korea; tkim8@ncsu.edu; 3 Joint Department of Biomedical Engineering, The University of North Carolina at Chapel Hill and North Carolina State University, Chapel Hill, NC 27599, USA; jkim50@ncsu.edu

**Keywords:** piezoelectric array sensor, elasticity sensor, force sensor, face-shear mode, PMN–PT

## Abstract

We present the development of a 6 × 6 piezoelectric array sensor for measuring elasticity and force. The proposed sensor employs an impedance measurement technique, sensing the acoustic load impedance of a target by measuring the electrical impedance shift of face-shear mode PMN–PT (lead magnesium niobate–lead titanate) single crystal elements. Among various modes of PMN–PT single crystals, the face-shear mode was selected due to its especially high sensitivity to acoustic loads. To verify the elasticity sensing performance, gelatin samples with different elastic moduli were prepared and tested. For the force measurement test, different magnitudes of force were loaded to the sensing layer whose acoustic impedance was varied with applied forces. From the experimental results, the fabricated sensor showed an elastic stiffness sensitivity of 23.52 Ohm/MPa with a resolution of 4.25 kPa and contact force sensitivity of 19.27 Ohm/N with a resolution of 5.19 mN. In addition, the mapping experiment of elasticity and force using the sensor array was successfully demonstrated.

## 1. Introduction

The tactile or haptic sensation is one of the most important sensory systems to perceive external stimuli, such as the elastic property of objects or contact force of hands [1]. The elasticity and the force measurements, therefore, are essentially required for a broad range of applications. In the surgery field, the elasticity/force measurement technique has been intensively researched for minimally invasive surgery (MIS) [2,3]. In open surgery, the surgeon can control the required force to grab tissues safely without damaging them and also separate the target tissue from other tissues based on the elasticity and force feedback [4]. In MIS, however, due to the small incision, the surgeon is not permitted to access the operating area directly and this can lead to unexpected errors and can endanger the patient’s safety [5,6]. In the biomedical field, elasticity sensors have been developed for characterizing the elastic property of human ovum and for sensing the intraocular pressure (IOP) in the human eye [7,8,9]. Tonometry is a traditional method to measure eye pressure which is performed on the cornea. This method estimates the elastic property of the ovum indirectly through the correlation between the applied pressure and the corneal thickness, which is not suitable for self-administration by patients [10]. For health care and service robotics applications, tactile sensor arrays have been widely used as an artificial skin for humanoid robots [11,12]. The artificial skin needs to be able to sense various tactile sensory modalities including contact force, stiffness, texture, and temperature. Therefore, it remains a challenge to develop the multi-functional sensor system for the integration in the artificial sensing device [13,14]. The elasticity sensors have also been developed for the muscle stiffness measurement in physical human-robot interaction research. [15]. Most muscle stiffness sensors estimate the stiffness by measuring skin displacement induced by the constant pressure, which may cause inconvenience and discomfort to patients [16].

Various electromechanical sensors including piezoresistive, capacitive, and piezoelectric sensors have been developed for the elasticity and force measurement [17,18,19,20]. Piezoresistive-type sensors based on the silicon device are widely used in robotics and medicine fields because of the simple working principle and the low crosstalk noise compared to the capacitive sensors [21,22,23]. Recently, piezoresistive pressure sensors based on conductive polyurethane sponges with carbon nanotubes and graphene also have been researched for applications such as wearable devices and artificial intelligence due to being lightweight, flexible, and having high sensitivity [24,25]. The capacitive-type sensor consisting of two electrodes is another popular device due to its highly sensitive frequency response and a wide dynamic range. This type of sensor is also suitable for large-area sensing applications with high spatial resolution [26,27,28]. The piezoelectric sensors using polyvinylidene fluoride (PVDF) films based on the direct piezoelectric effect are also widely used in the biomedical field because of the high sensitivity, flexibility, and inertness to chemical agents [29,30,31]. However, those types of sensors mentioned above usually measure the elasticity and the force separately and indirectly, which is likely to be complicated and time-consuming. For example, typical elasticity sensors estimate the elasticity indirectly through the relationship between the applied force and the deformation [31,32,33,34].

In this paper, we present a piezoelectric array sensor capable of measuring both the elasticity and force directly. Basically, the sensor employs the acoustic impedance measurement technique with a face-shear mode PMN–PT (lead magnesium niobate–lead titanate) and other single crystals [35,36], which was previously introduced by our group for sensing and actuation [37,38,39,40]. The elastic compliance of the PMN–PT crystal is about 6 times higher compared to PZT–5H and this offers decreased sensor size for a given operating frequency. The electromechanical coupling coefficient of PMN–PT is also higher (>0.9) than that of PZT-5H (>0.75) which leads to a broader operating bandwidth [41,42]. In addition, the PMN–PT piezoelectric sensor can provide enhanced overall performance due to its extremely high piezoelectric coefficient [43,44]. The prototyped array-type elasticity/force sensor in this study was able to sense Young’s modulus directly, which was an obvious measure of the elastic property of a target material. The sensor also simply detected applied external force by utilizing the acoustic impedance shift of the sensing layer resulting from the nonlinear elastic effect (also known as the strain hardening effect) [45].

## 2. Sensor Modeling

### 2.1. Elasticity Sensing Model

**Figure 1:**
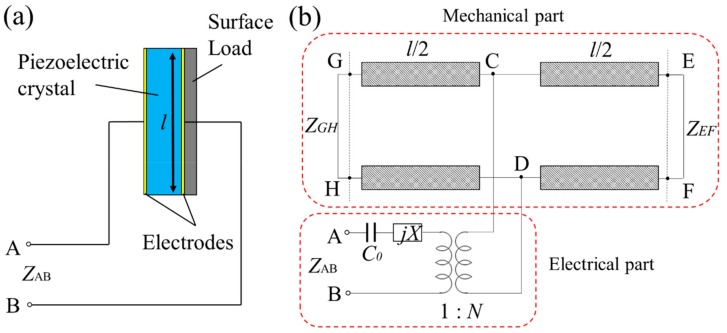
(**a**) A schematic of surface load sensing model; (**b**) an equivalent circuit model of the piezoelectric crystal with the acoustic loads where *X* is the reactance of the equivalent circuit and *N* is the turs ratio of a transformer.

The proposed piezoelectric sensor was designed for both elasticity and force-sensing. A face-shear mode PMN–PT was selected for a sensing crystal due to its exceptionally high sensitivity to the acoustic load impedance [37,38]. The elasticity of objects is a function of the elastic properties such as Young’s modulus and shear modulus. These elastic moduli are directly related to its characteristic acoustic impedance. Hence, measuring the acoustic impedance of an object simply results in its elastic properties. The elastic modulus (*E*) can be expressed by [46]

(1) $$E=\rho v^{2}=\frac{Z_{L}^{2}}{\rho },$$

where *Z*_L_ is the acoustic impedance, *ρ* is the density, and *v* is the sound speed. As shown in Equation (1), the elastic modulus depends on the square of the acoustic load impedance. This relation supports the hypothesis that the acoustic impedance can be a sensitive parameter in determining the object’s elastic property. For elasticity sensing, the surface load sensing model studied in the previous work can be used [37,38]. A schematic of the surface load sensing model is shown in Figure 1a. In this model, the piezoelectric crystal with the acoustic loads can be expressed as an equivalent circuit model that has two mechanical ports and one electrical port, as shown in Figure 1b. Thus, the output electrical impedance (*Z*_AB_) of the sensor is

(2) $$Z_{\mathrm{AB}}=\frac{1}{j\omega C_{0}}\left(1+\frac{e_{36}^{2}/\varepsilon_{33}^{S}c_{66}}{\omega l\sqrt{\frac{\rho }{c_{66}}}}\frac{j(Z_{\mathrm{EF}}+Z_{\mathrm{GH}})Z_{C}\sin\omega l\sqrt{\frac{\rho }{c_{66}}}-2Z_{C}^{2}(1-\cos\omega l\sqrt{\frac{\rho }{c_{66}}})}{(Z_{C}^{2}+Z_{\mathrm{EF}}Z_{\mathrm{GH}})\sin\omega l\sqrt{\frac{\rho }{c_{66}}}-j(Z_{\mathrm{EF}}+Z_{\mathrm{GH}})Z_{C}\cos\omega l\sqrt{\frac{\rho }{c_{66}}}}\right).$$

Here, *ω* is the angular frequency, *C*_0_ is the clamped capacitance of the sensing element, *e*_36_ is the face shear mode (or 36 mode) piezoelectric stress constant, $\varepsilon_{33}^{S}$ is the clamped dielectric constant along the *z* axis, $c_{66}$ is the shear elastic stiffened constant, *l* is the crystal length, and *Z*_C_ is the acoustic impedance of the piezoelectric element. *Z*_EF_ and *Z*_GH_ are the acoustic load impedance from the mechanical port EF and GH, respectively. In the elasticity sensing, the port EF and the port GH can be considered as the acoustic impedances at the backing layer (*Z*_B_) and the loaded surface of the sensor (*Z*_L_), respectively. Thus, Equation (2) can be simplified as

(3) $$Z_{\mathrm{AB}}=\frac{1}{j\omega C_{0}}\left(1+\frac{k_{36}^{2}}{\beta l}\frac{j(Z_{B}+Z_{L})Z_{C}\sin\beta l-2Z_{C}^{2}(1-\cos\beta l)}{(Z_{C}^{2}+Z_{B}Z_{L})\sin\beta l-j(Z_{B}+Z_{L})Z_{C}\cos\beta l}\right),$$

where *k* is the electromechanical coupling coefficient (*k* = $e_{36}^{2}$/($\varepsilon_{33}^{S}$*c*_66_)) and *β* is the wave number (= *ω*/($c_{66}^{D}$/*ρ*)^1/2^). As shown in Equation (3), for the constant initial conditions of the sensor, the electrical impedance (*Z*_AB_) depends only on the external acoustic load impedance (*Z*_L_). By combining Equations (1) and (3), the sensor output (*Z*_AB_) can be expressed as a function of the elastic modulus of targets as follows:

(4) $$Z_{\mathrm{AB}}=\frac{1}{j\omega C_{0}}\left(1+\frac{k_{36}^{2}}{\beta l}\frac{j(Z_{B}+\sqrt{E\rho })Z_{C}\sin\beta l-2Z_{C}^{2}(1-\cos\beta l)}{(Z_{C}^{2}+Z_{B}\sqrt{E\rho })\sin\beta l-j(Z_{B}+\sqrt{E\rho })Z_{C}\cos\beta l}\right)$$

or

(5) $$Z_{\mathrm{AB}}=f(E).$$

Equation (5) simply implies that the elastic modulus of objects (*E*) can be obtained by measuring the electrical impedance of the piezoelectric sensor (*Z*_AB_).

### 2.2. Force-Sensing Model

For force-sensing, an additional elastic layer (e.g., PDMS or polydimethylsiloxane) on the front surface of the piezoelectric crystal is required. Soft solid materials such as rubber have nonlinear elastic properties so that their elasticity varies with external loads because of the cross-linking system [47]. Thus, when the external force is applied to the top surface of the sensor, the elastic modulus of the stressed layer changes [48]. This change leads to the variation in the acoustic impedance according to Equation (1). Assuming that the layer material behavior is incompressible, the density remains constant [49]. As a result, the external force can be measured from the sensing layer’s acoustic impedance change by means of changes in the electrical impedance of the piezoelectric sensor. The nonlinear elasticity of sensing layers plays an important role in this force-sensing mechanism. Thus, the sensing layer’s nonlinear elasticity should be characterized first. There are a number of ways to explain the nonlinear elastic property of polymer materials such as the neo-Hookean model [50], second-order Ogden model [51], and the third-order Mooney–Rivlin model [52]. The Mooney–Rivlin model was chosen in this study due to its simplicity and accuracy for rubber materials. The Mooney–Rivlin equation can be expressed by as [49,53]

(6) $$W=C_{1}[\lambda^{2}+2{\left\{1-\nu (\lambda -1)\right\}}^{2}-3]+C_{2}[2\lambda^{2}{\left\{1-\nu (\lambda -1)\right\}}^{2}+{\left\{1-\nu (\lambda -1)\right\}}^{4}-3],$$

where *W* is the stored strain energy density, *C*_1_ and *C*_2_ are the constants whose unit is the same as that of stress, *λ* is the draw ratio along the edge of the material and *v* is the Poisson ratio. Considering the relationship between the strain energy and the stress, the strain energy can be defined by

(7) W=∫σdε.

Thus, stress can be expressed as

(8) $$\begin{array}{l} \sigma =\frac{dW}{d\varepsilon }=C_{1}\left(-2(1-\varepsilon )+\frac{4\nu }{{\left(1-\varepsilon \right)}^{2}}\right)+\\ C_{2}\left(4\nu -4(1-\varepsilon )\left(1-2\left(1-\frac{1}{1-\varepsilon }\right)\nu \right)+\frac{4\nu \left(1-2\left(1-\frac{1}{1-\varepsilon }\right)\nu \right)}{{\left(1-\varepsilon \right)}^{2}}\right) \end{array}$$

where *σ* is the stress and *ε* is the strain. The constants *C*_1_ and *C*_2_ can be estimated from a Mooney–Rivlin plot, (*σ*/*λ*−1/λ_2_ versus 1/*λ*) [53]. The elastic modulus (*E*) can be defined as

(9) $$E=\frac{\partial \sigma }{\partial \varepsilon },$$

From Equations (8) and (9), *E* can be rewritten by

(10) $$\begin{array}{l} E=C_{1}\left(2+\frac{8\nu }{{\left(1-\varepsilon \right)}^{3}}\right)+\\ C_{2}\left(-\frac{8\nu }{1-\varepsilon }+\frac{8\nu^{2}}{{\left(1-\varepsilon \right)}^{4}}+4\left(1-2\left(1-\frac{1}{1-\varepsilon }\right)\nu \right)+\frac{8\nu \left(1-2\left(1-\frac{1}{1-\varepsilon }\right)\nu \right)}{{\left(1-\varepsilon \right)}^{3}}\right) \end{array}$$

or

(11) E=g(ε).

As the strain (*ε*) is directly related to the stress (*σ*) which is defined as the average force (*F*) per unit area (*A*), the elastic modulus (*E*) can also be written as

(12) E=h(F).

Finally, the force-sensing model can be obtained by substituting Equation (10) into Equation (4):

(13) $$Z_{\mathrm{AB}}=\frac{1}{j\omega C_{0}}\left(1+\frac{k_{36}^{2}}{\beta l}\frac{j\left(Z_{B}+\sqrt{E(F)\rho }\right)Z_{C}\sin\beta l-2Z_{C}^{2}(1-\cos\beta l)}{\left(Z_{C}^{2}+Z_{B}\sqrt{E(F)\rho }\right)\sin\beta l-j\left(Z_{B}+\sqrt{E(F)\rho }\right)Z_{C}\cos\beta l}\right)$$

or

(14) $$Z_{\mathrm{AB}}=k(F).$$

Similar to the elasticity sensing model, Equation (14) implies that the force (*F*) can be sensed by measuring the electrical impedance of the piezoelectric sensor (*Z*_AB_).

## 3. Sensor Design and Experimental Methods

### 3.1. Sensor Array Fabrication

**Figure 2:**
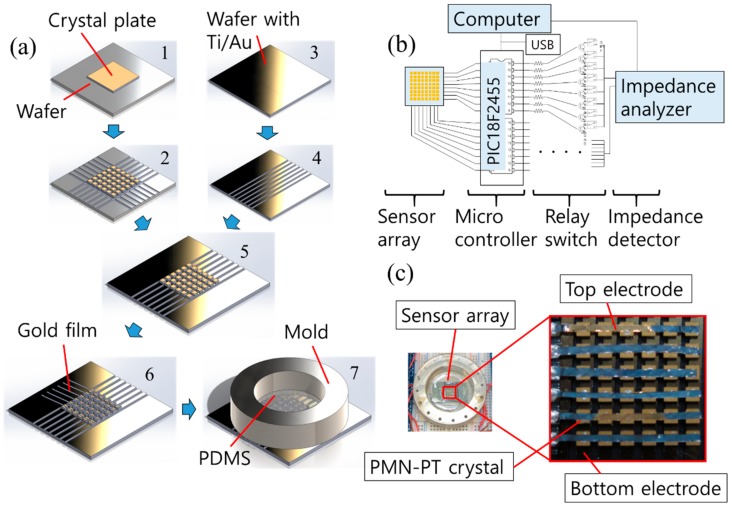
(**a**) 6 × 6 array fabrication process; (**b**) the block diagram of the array impedance measurement system and the switch circuit; (**c**) the fabricated 6 × 6 array with the switch and microcontroller circuits.

The designed sensor consisted of a 6 × 6 array of face-shear mode PMN–PT sensing elements with 1MHz operational frequency. As a sensing layer, PDMS was used because of its transparency, simple fabrication, and biocompatibility. The design specification of the sensor was summarized in Table 1. Figure 2a shows the overall fabrication process for the designed sensor. The face-shear mode PMN–PT crystal plate (10 × 10 × 0.5 mm^3^) was prepared first and electrodes (Ti/Au, 10/50 nm) were applied to both large surfaces for electrical connections. The supporting wafer then bonded to the backside of the crystal plate using a bonding wax (1). The crystal was diced into 36 elements with a pitch of 1.3 mm (2). For the bottom electrode connection, a silicon wafer with Ti/Au electrode was prepared (3) and partially diced (4). The crystal plate array (2) was bonded to the diced silicon wafer (4) with epoxy (Epotek 301, Epoxy Technology, Inc.). The supporting wafer was detached from the array at the melting temperature (70 °C) of bonding wax (5). For the top electrode connection, gold-coated thin films (0.4 mm in width) were bonded to the top surface of the array using silver epoxy (6). Then, a PDMS (Sylgard 184, Dow Corning Corp.) layer was applied to the crystal array through a circular-shaped mold as a sensing/protecting layer (7). Each element of the crystal array was connected to both the row (top) electrode and the column (bottom) electrode line. Finally, the fabricated sensor was connected to an impedance analyzer (HP 4294A, Agilent) through an 8-channel relay board (5 V-relay module, SainSmart) with a microcontroller (PIC18F2550, Electronics-DIY). The impedance variance of all crystal elements was scanned one by one followed by data acquisition. Figure 2b,c show the block diagram of the measuring system and the photograph picture of the fabricated sensor, respectively.

### 3.2. Experimental Method

**Figure 3:**
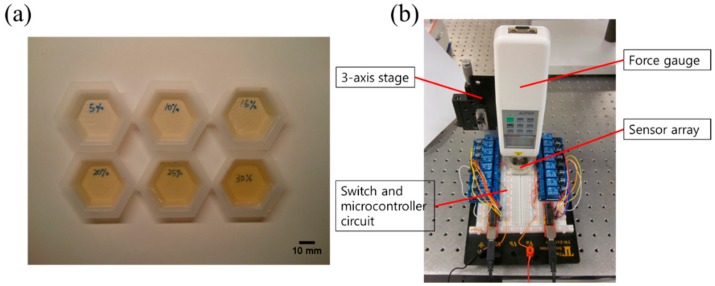
(**a**) The fabricated test samples with different weight ratios; (**b**) The experimental setup for the stress-strain measurement and force-sensing test.

For the test sample material, gelatin powders (Great Lakes Gelatin Company, IL) were used since its elastic stiffness is efficiently controlled by the adjustment of weight ratio (WR) of powder. The test samples were prepared using the traditional fabrication method for tissue-mimicking phantoms which was referred to in published papers [54,55]. Gelatin powders with 5% to 30% WR were blended in the warm water (65 °C). Then, gelatinous samples were degassed in a vacuum chamber and stored in a refrigerator for 3 h. Figure 3a shows the fabricated test samples with different WR. To characterize the sample’s elastic modulus as reference data, the force-deformation method [56] was used. Young’s modulus of each sample can be obtained according to Equation (15):

(15) $$E=\frac{\sigma }{\varepsilon }=\frac{F/A_{0}}{\Delta L/L_{0}},$$

where *ε*, *σ*, *E*, *F*, *A*_0_, Δ*L*, and *L*_0_ are the induced strain, the applied stress, Young’s modulus, the applied normal force, the initial effective area, the change in thickness, and the original thickness of the sample, respectively. The shear modulus of the sample can be calculated using Equation (16):

(16) $$G=\frac{E}{2(1+\nu )},$$

where *G* and *v* are the shear modulus and the Poisson’s ratio of the gelatin sample. In this study, a Poisson’s ratio of 0.5 was used, which was a reasonable value for gelatin samples [18]. To verify the sensing performance, we tested the bulk crystal without dicing the crystal into individual pieces first. Fabricated gelatin samples were attached to bulk crystals (10 mm × 10 mm × 1 mm), and the electrical impedance spectra of the crystals were measured. Then, to verify the array sensing ability, the gelatin sample was positioned on the center of the array, and the electrical impedances of all array elements were measured. For the force-sensing test, a stress-strain curve of the sensing layer was obtained using the force-deformation method to characterize its nonlinear elastic property. Then, similar to the elasticity test, both the bulk and the array sensing experiment were performed for the force-sensing. The normal force (0.1 N to 5 N) was applied to the top of the bulk and array sensor through the tip of a force gauge (HF-10, ALIYIQI). The induced deformation of the sensing layer was read through the scale on the *y*-axis of the stage with a displacement resolution of 1 μm. The electrical impedance shift (Δ*Z*_AB_) at the resonance of the bulk crystal and all 36 elements was measured three times and averaged using the impedance analyzer. According to Equation (13), Δ*Z*_AB_ can be converted to the elastic modulus shift (Δ*E*) of the sensing layer. Then, the induced strain and stress can be obtained from Equations (8) and (10). Finally, the applied force can be calculated using the applied stress to the sensing layer. The force-sensing test setup for the array sensor is shown in Figure 3b.

## 4. Results and Discussion

### 4.1. Elasticity Sensing Test Results

**Figure 4:**
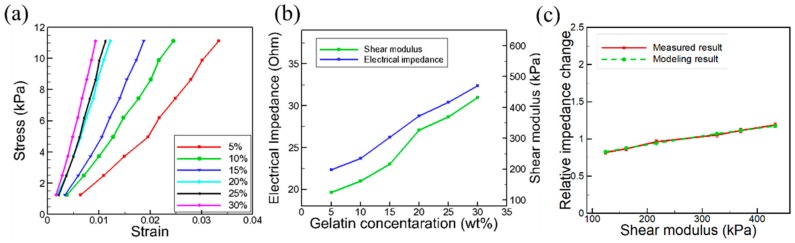
(**a**) The stress-strain measurement result for test samples with different weight ratios; (**b**) the measured electrical impedance and shear modulus of as a function of gelatin concentration; (**c**) the measured and calculated relative electric impedance shift of the bulk crystal. The calculated result was obtained from Equation (4).

The stress-strain measurement result of gelatin samples with different WR is shown in Figure 4a. The force-sensing test setup was also used for the stress-strain measurement test of gelatin samples. When the applied stress increased, the strain also increased. The slope represented Young’s modulus of the gelatin sample, and it depended on the composition of each sample or the gelatin WR. The calculated results using Equations (15) and (16) for shear modulus of samples with the different WR were shown in Figure 4b. It was shown that the shear modulus increased almost linearly from 124 kPa to 432 kPa, as the gelatin WR increased from 5% to 30%. The bulk crystal test result with the different WR samples and the modeling result calculated from Equation (4) were compared as can be seen in Figure 4c. The measured electrical impedance shift was about 45 ± 2.5%, which shows an excellent (>98%) agreement with the calculated result. Although the equation (4) shows a nonlinear relation between the elasticity value and the output impedance, the given shear modulus range (100–500 kPa) resulted in the quasilinear trend.

**Figure 5:**
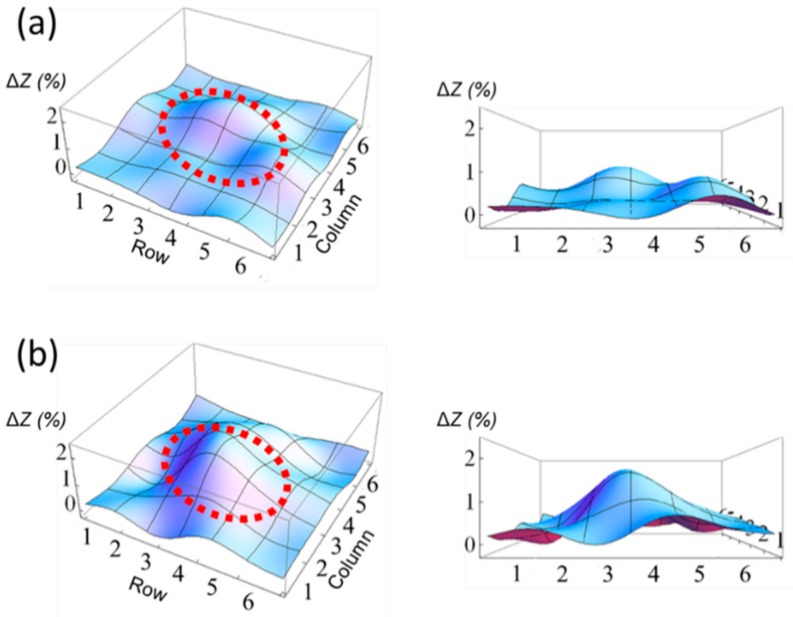
Elasticity sensing test results using the 6 × 6 sensor array for (**a**) 5% WR gelatin sample and (**b**) 30% WR gelatin sample. Red dotted circles represent the position of each gelatin sample.

Figure 5 shows the result for the array sensing test with different WR of gelatin samples (5% and 30%). Each sample was loaded to the center of the array, and the electrical impedances of all array elements were measured. Red dotted circles indicate the loaded position on the array. The largest electrical impedance shift was found to be 1.50 ± 0.04% from 30% WR sample while the smallest shift was found to be 0.63 ± 0.04% from 5% WR sample. The corresponding shear modulus was 124 kPa and 432 kPa for 5% and 30% WR samples, respectively. These results indicate the acoustic impedance variation of elastic objects can be directly measured using the proposed sensing technique. There was a difference in measured electrical impedance values between the bulk crystal and the array sensor under the same acoustic loads. One possible reason is that the enclosed PDMS of array elements acts as acoustic loads, which can dampen surface vibrations of sensing elements, and thus, reduces the acoustic load sensitivity. Nevertheless, more than two times higher electrical impedance shift was observed from 30% WR sample (1.5%) compared with 5% WR sample (0.63%), showing the possibility of the proposed sensor array for the elasticity sensing applications.

### 4.2. Force-Sensing Test Results

**Figure 6:**
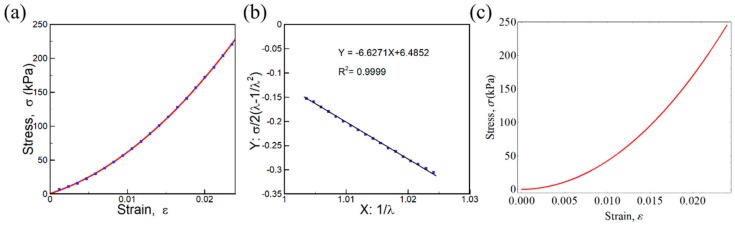
(**a**) The measured stress-strain diagram of polydimethylsiloxane (PDMS) sensing layer; (**b**) calculated Mooney–Rivlin plot (σ/λ −1/λ_2_ verses 1/λ); (**c**) the calculated stress-strain diagram of PDMS using the third-order Mooney model from Equation (10).

The measured stress-strain diagram of PDMS based on the force and deformation method is shown in Figure 6a. It was evident that the slope of the curve (= Young’s modulus) increased as the stress increased. This nonlinear nature of the sensing layer is a crucial factor for the force-sensing mechanism. For the nonlinear model calculation, the constants *C*_1_ (= −6.6271) and *C*_2_ (= 6.4852) were obtained from the linear fitted curve in the Mooney–Rivlin plot, as shown in Figure 6b. The calculated stress-strain diagram of PDMS using the third-order Mooney model is presented in Figure 6c. The model result agreed well with the measured stress-strain diagram (Figure 5a).

**Figure 7:**
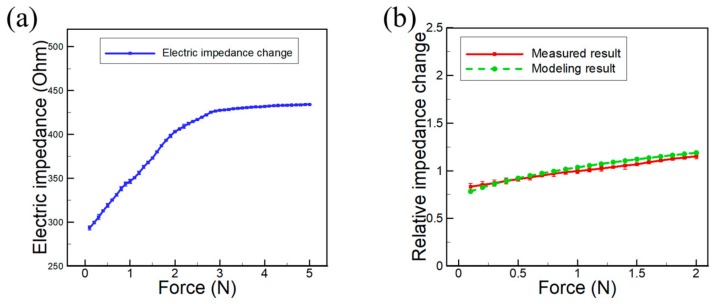
(**a**) Force-sensing test results for bulk crystals with an external force ranging from 0.1 N to 5 N; (**b**) the measured and calculated relative electric impedance shift of the bulk crystal as a function of applied force. Equation (13) was used for the calculated result.

The force-sensing test results using bulk crystals are shown in Figure 7a. The normal force (0.1 N to 5 N) was applied to the PDMS sensing layer on the bulk crystal, and the electrical impedance shift was measured. The electrical impedance rapidly increased up to 2 N, but after this point, the increment was reduced to almost zero, which was because that the sensing layer was totally hardened or stiffened and no longer able to change upon external forces. The measured and calculated (using Equation (13)) relative impedance shift curves as a function of applied forces are shown in Figure 7b, which shows good agreement (>95%) to each other.

**Figure 8:**
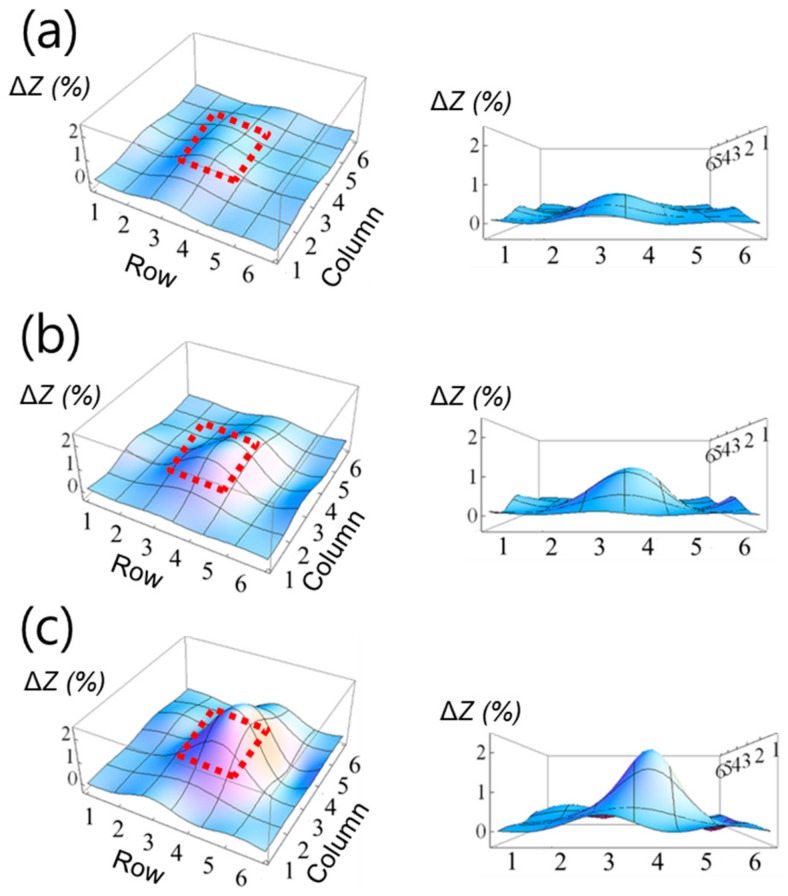
Force-sensing test result using the 6 × 6 sensor array; (**a**) 2 N (**b**) 3 N, and (**c**) 4 N forces were applied. Red dotted squares indicate the area over which forces were applied.

Figure 8 presents the force-sensing results using the array sensor. Red dotted squares indicate the areas over which forces were applied. The amount of the electrical impedance shift of loaded elements increased with the applied forces. Specifically, 0.43 ± 0.07%, 0.87 ± 0.02%, and 1.56 ± 0.10% of electrical impedance shifts were found for 2 N, 3 N, and 4 N loaded sensor arrays, respectively. These shifts were smaller than those of the bulk crystal sensing results (37.48 ± 0.55%, 45.89 ± 0.51%, and 47.44 ± 0.52%) due to the damping effect, similar to the case of the elasticity sensing result. The force-sensing test results suggest that the prototyped sensor array can be promising for the external force-sensing as well as elasticity sensing. For the sensing layer, a 10:1 mixture ratio of pre-polymer and curing agent (or cross-linking agent) was applied in this study. Considering the sensor performance, the softer sensing layer can be applied for higher sensitivity. For example, PDMS with 30:1 ratio has much lower elastic modulus (*E* < 0.42 MPa) than that of PDMS with 10:1 ratio (*E* < 2.04 MPa) [57]. In contrast, the harder sensing layer can be more suitable for the application requires a broad dynamic range. Thus, the stiffness of the sensing layer should be optimized for the specific application because it is one of the most important factors that determines the sensor sensitivity and dynamic range. The force-sensing test results suggest that the prototyped sensor array can be promising for the external force-sensing purpose as well as the elasticity-sensing application. The sensor specification based on the test results for the proposed sensor array is summarized in Table 2.

## 5. Conclusions

In conclusion, we developed a piezoelectric array sensor using face-shear mode PMN–PT crystals for elasticity and external force-sensing. This study demonstrated that the loaded sample’s elastic properties and the magnitude of the applied force are measured by collecting the electric impedance of the sensor array. As the test results show acceptable agreement with the theoretical model results, we expect that this sensor design is easily modified and optimized for advanced tactile sensing applications such as MIS tools, artificial skin for surgical robotics, and various bio-imaging systems.

## Figures and Tables

**Table 1:** Specification for the fabricated 6 × 6 sensor array.

Sensing Element #	Array Dimensions	Element Dimensions	Sensing Crystal	Sensing Layer	Operational Frequency
36	9 × 9 × 1 mm^3^	800 × 800 × 500 μm^3^	Face-shear mode PMN–PT	PDMS	1 MHz

**Table 2:** Sensing performance comparison for the proposed sensor.

	Proposed Sensor (Piezoelectric Type)		Compared Sensor * (Capacitive Type)	
Performance	Elasticity Sensing	Force-Sensing	Elasticity Sensing	Force-Sensing
Range	~432 kPa	~1 N	0.7–1.2 MPa	0–0.5 N
Sensitivity	23.52 Ohm/MPa	19.27 Ohm/N	-	-
Resolution	4.25 kPa	5.19 mN	0.1 MPa	0.2 mN

* Peng et al. [33].
